# Bedfordshire Hospital Trained Nurses' Institute

**Published:** 1893-02-11

**Authors:** 


### BEDFORDSHIRE HOSPITAL TRAINED NURSES' INSTITUTE.

I notice that your "Special Com.," in your issue of the 4th inst., writes of this institution in the following terms: "There can be no doubt that it would be most desirable to attach the Nurses' Institute to the Infirmary, and to make arrangements that its board should train the nurses there. This would give a much better guarantee of efficiency to those who require the services of a private nurse." In order to prevent any misunderstanding on the part of the public, and in justice to my own nurses who seem to be depreciated, I trust you will permit me to say, through your columns, that all my nurses are certificated, and have been fully and systematically trained in the large hospitals, most of them in London, with all the advantages of lectures, examinations, and varied experience; they have all been most carefully selected by me, who am myself a trained nurse, and I have found them a highly efficient staff, and I may add that their services are warmly appreciated by the public in Bedford and the neighbourhood.

(Signed) K. Rawson, Hon. Lady Superintendent.

*** We made no reflection, and intended no reflection, upon the nurses at present employed, who, we are ready to believe, deserve all the kind things our correspondents say of them. Our Commissioner was, however, contending for a sound principle—that every provincial hospital should train a staff of nurses for the use and comfort of the private patients of the district which it serves. This system will ensure that everybody able and willing to pay a trained nurse shall be able to secure her services on application to the hospital which is nearest to the patient's own dwelling. Until this is done, experience proves that many evils, otherwise remedial, must prevail.—Ed. T. H.

